# Humans can detect axillary odor cues of an acute respiratory infection in others

**DOI:** 10.1093/emph/eoad016

**Published:** 2023-05-27

**Authors:** Arnaud Tognetti, Megan N Williams, Nathalie Lybert, Mats Lekander, John Axelsson, Mats J Olsson

**Affiliations:** Division of Psychology, Department of Clinical Neuroscience, Karolinska Institutet, Stockholm, Sweden; CEE-M, CNRS, INRAE, Institut Agro, University of Montpellier, Montpellier, France; Division of Psychology, Department of Clinical Neuroscience, Karolinska Institutet, Stockholm, Sweden; Division of Psychology, Department of Clinical Neuroscience, Karolinska Institutet, Stockholm, Sweden; Division of Psychology, Department of Clinical Neuroscience, Karolinska Institutet, Stockholm, Sweden; Stress Research Institute, Department of Psychology, Stockholm University, Stockholm, Sweden; Osher Center for Integrative Health, Department of Clinical Neuroscience, Karolinska Institutet, Stockholm, Sweden; Division of Psychology, Department of Clinical Neuroscience, Karolinska Institutet, Stockholm, Sweden; Stress Research Institute, Department of Psychology, Stockholm University, Stockholm, Sweden; Division of Psychology, Department of Clinical Neuroscience, Karolinska Institutet, Stockholm, Sweden

## Abstract

**Background and objectives:**

Body odor conveys information about health status to conspecifics and influences approach-avoidance behaviors in animals. Experiments that induce sickness in otherwise healthy individuals suggest that humans too can detect sensory cues to infection in others. Here, we investigated whether individuals could detect through smell a naturally occurring acute respiratory infection in others and whether sickness severity, measured via body temperature and sickness symptoms, was associated with the accuracy of detection.

**Methodology:**

Body odor samples were collected from 20 donors, once while healthy and once while sick with an acute respiratory infection. Using a double-blind, two-alternative forced-choice method, 80 raters were instructed to identify the sick body odor from paired sick and healthy samples (i.e. 20 pairs).

**Results:**

Sickness detection was significantly above chance, although the magnitude of the effect was low (56.7%). Raters’ sex and disgust sensitivity were not associated with the accuracy of sickness detection. However, we find some indication that greater change in donor body temperature, but not sickness symptoms, between sick and healthy conditions improved sickness detection accuracy.

**Conclusion and implications:**

Our findings suggest that humans can detect individuals with an acute respiratory infection through smell, albeit only slightly better than chance. Humans, similar to other animals, are likely able to use sickness odor cues to guide adaptive behaviors that decrease the risk of contagion, such as social avoidance. Further studies should determine how well humans can detect specific infections through body odor, such as Covid-19, and how multisensory cues to infection are used simultaneously.

## INTRODUCTION

Pathogens have exerted strong selection pressure on animal populations, including humans, throughout evolutionary history, evident by the complex immune systems many organisms have evolved as antiparasitic defense systems (for reviews see [1, 2]). While the human immune system is highly effective at detecting and eliminating harmful pathogens from the body, activation of the immune response is metabolically costly and can involve sickness behaviors and symptoms, such as fatigue, loss of appetite, and fever, that are further incapacitating and potentially fitness reducing (for reviews see [2–4]). Thus, evolutionary theorists suggest additional behavioral defenses against disease evolved to complement the immune system by preventing contact with infectious agents in the first place. This proactive behavioral defense system contains mechanisms for detecting sickness cues in others, which can help launch preparatory immune responses [5–8] and facilitate adaptive behavioral responses, such as the avoidance of potentially contagious conspecifics (for reviews see [1, 9]).

In fact, there is evidence to suggest that cues of sickness are detectable in human faces. For example, studies that experimentally induce sickness via injection with an endotoxin (lipopolysaccharide, LPS) demonstrate that within a couple of hours, the faces of sick individuals have paler skin and lips, drooping eyelids and sagging corners of the mouth [10, 11]. Similarly, evidence indicates that body odor also conveys cues to health status in humans [12–14]. Body odor is comprised of low-weight molecular compounds that evaporate easily called volatile organic compounds (VOCs) [15]. VOCs are emitted constantly from the body because all organs produce cellular VOCs as a metabolic byproduct. After entering the blood stream, VOCs are released through breath, saliva, urine, feces, the skin and blood [16]. When sick, individuals may produce novel VOCs or different expression patterns and ratios of VOCs. Accordingly, changes in human body odor may indicate a disease state. Indeed, historical texts illustrate that since the time of Hippocrates smell has been used by medical practitioners in disease detection. Even today, a sweet fruity or a strong musty odor from patients’ breath is taken as an indicator of diabetic ketoacidosis or advanced liver disease, respectively [17]. Furthermore, observational data suggests a relationship between various sources of body odor (e.g. breath, sweat, and urine), and several diseases, including cholera, pneumonia, tuberculosis, and smallpox (for reviews see 18, 19]). Moreover, modern advances in chemical analysis (e.g. GC/MS and electronic noses) confirm that diseases alter the VOCs released from the human body (e.g. [20]). For example, GC/MS analysis accurately distinguishes between the VOC expression patterns in breath samples from patients with Mycobacterium tuberculosis and healthy controls, corroborating historical observations that tuberculosis causes the breath to have a foul odor [19, 21]. Taken together, these findings suggest humans may be able to identify naturally occurring sickness in others via smell alone, but prior empirical research has primarily employed experimental sickness models (i.e. LPS).

For instance, Olsson and colleagues [12] collected axillary body odors samples from human participants that were experimentally made sick by an intravenous injection of LPS. ‘Sick’ samples were rated by others as smelling more aversive and less healthy than body odor samples from healthy participants. A similar experiment using the same sickness induction methodology found that while healthy human urine becomes less aversive smelling throughout the course of a day, ‘sick’ urine increases in aversiveness and exhibits an altered volatile composition [13]. Finally, other studies have shown that exposing participants to ‘sick’ body odors while they rated faces resulted in lower liking scores of faces, suggesting that olfactory cues to sickness are not only detectable but may also initiate adaptive social responses, such as avoidance [14, 22]. While these studies indicate that olfactory cues to an experimentally induced transient inflammatory response are perceptually detectable in humans, less is known about whether naturally occurring illnesses can be detected via smell.

Only a few studies have investigated the perceptions of body odors of individuals with naturally occurring contagious infections (e.g. [23, 24]). One such study found that body odors samples from male patients infected with gonorrhea were rated by women as smelling significantly less pleasant than samples from healthy controls and recently recovered patients [23]. In contrast to these significant findings, no significant differences were discovered between ratings of an individual’s body odor while healthy versus sick with an acute respiratory infection [24]. These conflicting results may be due to different infections, one viral and the other bacterial. Moreover, both studies were small, only 18 and 46 raters, respectively [23, 24], making it difficult to draw any robust conclusions about whether humans can use odor cues in identifying contagious individuals.

The present study builds on the prior study of acute respiratory infection, which was conducted by our research group, using the same body odor samples [24]. In Sarolidou, Tognetti et al. [24], participants rated body odor samples from individuals with an acute respiratory infection, one at a time on four separate dimensions: disgust, intensity, pleasantness and health. While sick body odor samples tended to be rated more disgusting, more intense, less pleasant and less healthy, these trends were not statistically significant. In the current study, we doubled the sample size of raters (*n* = 80) and used a more sensitive psychometric method to determine whether humans can identify through body odor individuals with an acute respiratory infection. We measured sickness detection by instructing participants to identify the sick body odor from within a pair of body odor samples. Each pair contained two samples from the same donor. One sample was collected while the donor was healthy, and the other sample was collected while the donor was sick with a respiratory infection.

Another aim of the present study was to investigate potential predictors affecting the detection of sick individuals, specifically changes in body temperature and sickness symptoms between healthy and sick conditions. Additionally, we examined whether rater sex and disgust sensitivity, measured via the Body Odor Disgust Scale (BODS) [25], were associated with sickness detection accuracy. Women perform better than men on measures of olfactory acuity (for review [26]) and exhibit greater disgust sensitivity, particularly to pathogen-connoting stimuli (e.g. [27], for review see [28]). Researchers suggest disgust is an evolved motivational system for disease avoidance (for review [29]). Thus, we predicted that women and individuals with higher disgust sensitivity would exhibit more accurate, or potentially more biased, sickness detection through smell.

In sum, the current study aimed to (i) investigate whether humans can discriminate between sick and healthy body odors using a naturally occurring rather than experimentally induced sickness model, (ii) examine whether overall changes in temperature between sick and healthy conditions and/or sickness symptoms could explain some of the variance between rater sickness detection scores for different body odor donors, and (iii) evaluate whether individual differences in sex, age and disgust sensitivity correlate with rater accuracy in detecting sick odors.

## METHODOLOGY

### Participants

#### Raters

Eighty-one participants were recruited for the body odor assessment portion of this study via the Karolinska Institutet’s online recruitment system and via posters displayed around campus. We refer to these participants as raters. To be eligible, raters could not be pregnant, had to be non-smokers, had to have normal or corrected-to-normal vision (tested with vision scales), had to have a functional sense of smell (self-report; ‘Do you have a normal sense of smell today?’), and needed to be 18 years of age or older. After excluding one participant for having a non-functional sense of smell, the final sample size was *n* = 80 raters (44 women, 36 men; mean age = 31.4 years, *SD* = 10.7, range = 18–64). Unfortunately, a power analysis was not performed prior to the collection of data. However, an effect size as small as *d* = 0.29 can be detected with 80 participants at 80% power with α = 0.05 (G*Power 3.1.9.4), based on the statistical analysis described below (i.e. Wilcoxon signed rank test). As compensation for their participation, raters received a gift card valued at 99 SEK. All participants completed and signed an informed consent form. The study was approved by the regional ethical review board of Stockholm (2017/55-31/4).

#### Donors

The odor samples used in the present study were collected in an earlier study examining the effects of acute respiratory infections on sleep [30]. That study was approved by the regional ethical review board of Stockholm (2011/2034-31/1). For more detailed information concerning the study protocol, see Lasselin et al. [30]. The odor samples used here were also previously used in another study by our group [24].

Body odor was sampled from *n* = 23 participants (14 women, 9 men; mean age = 32.4, *SD* = 13.3), referred to as donors, during two different conditions (sick and healthy) that were separated by 4 weeks. Having a respiratory infection was defined as having an acute respiratory infection or influenza-like illness, specifically, donors had to self-report at least one of the following respiratory symptoms: cough, sore throat, shortness of breath or coryza, as well as one of the following systemic symptoms: fever, headache, malaise or myalgia (see [30] for more details). The same day donors experienced their first sickness symptoms, they were provided a research kit that contained a digital in-ear thermometer (Thermoscan, Braun), and questionnaires where they recorded sickness symptoms and morning and evening body temperature for 7 days. During each condition, t-shirts (with nursing pads sewn into the armpits) were worn during sleep for two nights and stored in plastic bags during the day. On odor sampling days (prior to sleep), donors only cleaned themselves with perfume-free soap and shampoo (obtained by us) and were instructed to refrain from eating strong spices, garlic, or asparagus, as well as to avoid alcohol, smoke and perfumed products. Additionally, during the sick condition, donors were told to avoid using antipyretics or nasal sprays. Once research kits and t-shirts were returned to experimenters, all nursing pads were placed in glass jars and frozen at −35°C. After excluding two donors from analysis because of perfume use, and another donor for failure to return a t-shirt containing the nursing pads, the final number of donors was 20, each delivering 2 body odor samples (left and right armpit) for each condition (sick and healthy).

### Procedures

#### Chemosensory testing of raters

The test includes a total of 16 distinct concentrations of n-butanol. In our shortened version of the test, a single staircase approach was used starting from the middle, dilution step 8, rather than starting from the pens containing the lowest concentration of n-butanol (i.e. step 16). One reversal was considered sufficient to detect anosmia. That is, raters were first presented with the triplet of pens from dilution step 8. The participant then moved up or down in degrees of difficulty, reflected by the concentration of n-butanol, based on their ability to identify the odorous pen. Once three correct identifications were made, the triplet of pens corresponding to the concentration one dilution step above (weaker concentration) was presented until the first error was observed. Three correct identifications were used rather than the standard two for a more conservative measure since we used a shortened version of the Sniffin’ Sticks test (i.e. we attempted to increase the reliability of each step). If raters failed to distinguish the highest concentration of n-butanol (i.e. step 1), their threshold was determined to be above this concentration, and they were considered anosmic. The Sniffin’ Sticks results (see Supplementary Table SM1 for descriptive statistics) indicated that no raters were anosmic. However, one rater was excluded from the analysis because s/he reported not having a normal sense of smell that day.

#### Questionnaire to raters

Raters completed the Body Odor Disgust Scale (BODS), a 12-item measure used to assess individual differences in disgust sensitivity to various body odors (see Supplementary Table SM1 for descriptive statistics) [25]. Half the items measure sensitivity to one’s own body odors, while the other six items measure sensitivity to others’ body odors. In the current study, we only used the six questions pertaining to others’ body odors. These questions asked about raters’ experiences of different types of body odor (i.e. breath, urine, upper body sweat, feet, gas and feces) emanating from strangers in diverse contexts. Ratings were made on a 5-point Likert Scale ranging from 1 (not disgusting at all) to 5 (extremely disgusting). The BODS was administered between the first and second rounds of odor ratings (described below).

#### Ratings of odor samples

The odor rating portion of the experiment was administered in two rounds. During the first round, raters were presented with all paired odor samples in a unique randomized order; that is, 20 pairs consisting of one healthy and one sick body odor sample. Each sample contained both the left and right armpit pads. Before the presentation of odor pairs, an experimenter blind to the conditions removed jar lids and instructed raters to smell each jar once (max twice). Raters were further instructed not to touch the jars with their noses and not to blow into the jars. In addition, raters wore surgical gloves while handling jars. Raters were asked to identify which stimulus in a pair contained a sick body odor sample. Odor samples were always presented in opaque glass jars, and the position (left- or right-hand side) of the sick and healthy odor samples were randomized. A 20-s time interval was instituted between presentations of odor pairs to mitigate habituation effects (see [31]). After the first round of odor ratings, raters completed the BODS (see section ‘Questionnaires’). Then, raters began the second round of odor ratings, which was identical to the first round except odor samples were presented in a newly randomized order. Thus, in total (i.e. rounds 1 and 2 taken together) raters assessed 40 pairs of sick and healthy odor samples. This experiment was designed using PsychoPy 3.0 [32].

### Analyses

A two-sided Wilcoxon signed rank test with continuity correction, comparing sickness detection scores (i.e. the proportion of correct identifications) to chance level (50%), was performed to assess whether raters were able to detect sickness via odor.

To examine whether rater characteristics (i.e. sex and disgust sensitivity) influence rater detection of sickness via smell, we used a Bayesian Generalized Linear Mixed-Effects Model (bglmer function in the blme R package) with a binomial error structure. Our dependent variable was a correct response (i.e. binary response variable where 0 = incorrect identification of sick body odor and 1 = correct identification of sick body odor) by a rater for each of the 40 pairs of odor samples. Our explanatory variables were ‘BODS’ (i.e. Body Odor Disgust Scale score) and ‘Sex’. Additionally, we included a random intercept for each rater and donor (ID and ID_Donor, respectively). Thus, the model was:

Correct Response ~ Sex + BODS + (1 | ID) + (1 | ID_Donor)

Next, we used a similar Bayesian Generalized Linear Mixed-Effects Model to examine the influence of donor characteristics on rater sickness detection scores. We used separate models, one for each donor characteristic (i.e. explanatory variable).

**Figure 1.**
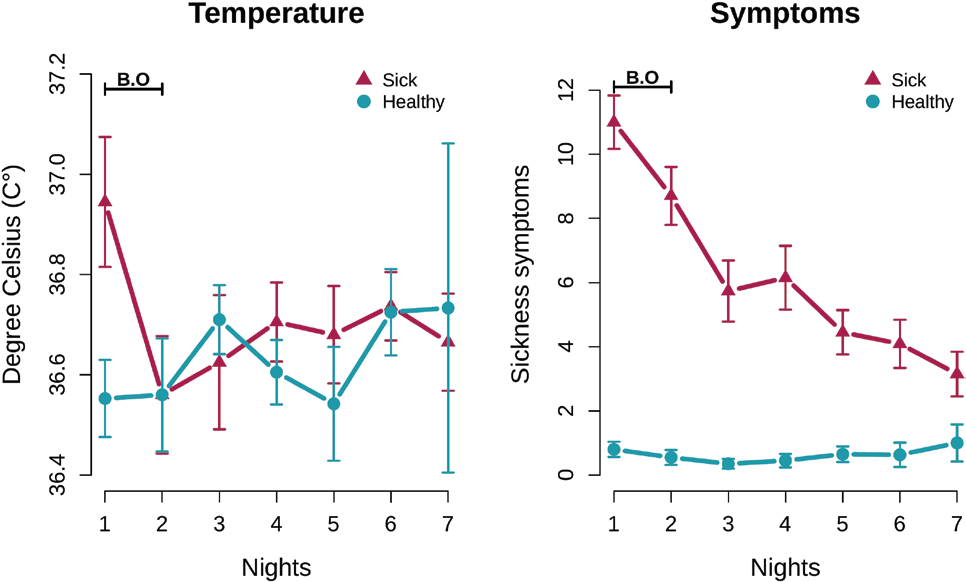
Donors’ body temperature and sickness symptoms measured over seven nights in both the sick and healthy conditions. Body odor was collected during nights 1 and 2 (denoted in figure by B.O.) in each condition. Ten sickness symptoms (i.e. sneezing, sore throat, fever, headache, congested nose, runny nose, cough, nausea, muscle pain, dizziness) were rated on a 4-point scale ranging from 0 = ‘none’ to 3 = ‘severe’, providing a total score of sickness symptoms ranging from 0 to 30. Error bars represent standard error

The first model looked at the change in donor temperature [Δ_temperature] between healthy and sick conditions on night 1 (see Supplementary Table SM2 for descriptive statistics on temperature change). While donor temperature was recorded for seven nights, body odor was only collected on nights 1 and 2 and we only find significant differences in body temperature between conditions on night 1 (two-sided Student’s *t*-test, *t* = 3.28, *df* = 18, *p* =.004; Fig. 1). Therefore, we used body temperature data from night 1 only. In addition, one donor failed to record body temperature on night 1, so only 19 donors were included in this model. The dependent variable was a correct response (i.e. binary response variable where 0 = incorrect identification of sick body odor and 1 = correct identification of sick body odor) by raters for a pair of odor samples. We also include a random intercept for each rater and donor, and random slopes for change in temperature by rater. Thus, the model was:

Correct Response ~ Δ_temperature + (1 | ID_Donor) + (1 + Δ_temperature | ID)

The second model looked at the change in donor sickness symptoms [Δ_symptoms] between healthy and sick conditions on night 1 (see Supplementary Table SM2 for descriptive statistics on number of symptoms). Again, sickness symptoms were recorded for seven nights but body odor was only collected on nights 1 and 2. For congruency with the temperature model, we again only used sickness symptom data recorded on night 1. The dependent variable was a correct response (i.e. binary response variable where 0 = incorrect identification of sick body odor and 1 = correct identification of sick body odor) by raters for a pair of odor samples. We also include a random intercept for each rater and donor, and random slopes for change in sickness symptoms by rater. Thus, the model was:

Correct Response ~ Δ_symptoms + (1 | ID_Donor) + (1 + Δ_symptoms | ID)

Statistical analyses were conducted using R, version 4.1.3. For the mixed models, the statistical significance of each variable was tested with likelihood ratio tests comparing the full model to those without the term of interest.

## RESULTS

### Sickness detection is above chance level

**Figure 2.**
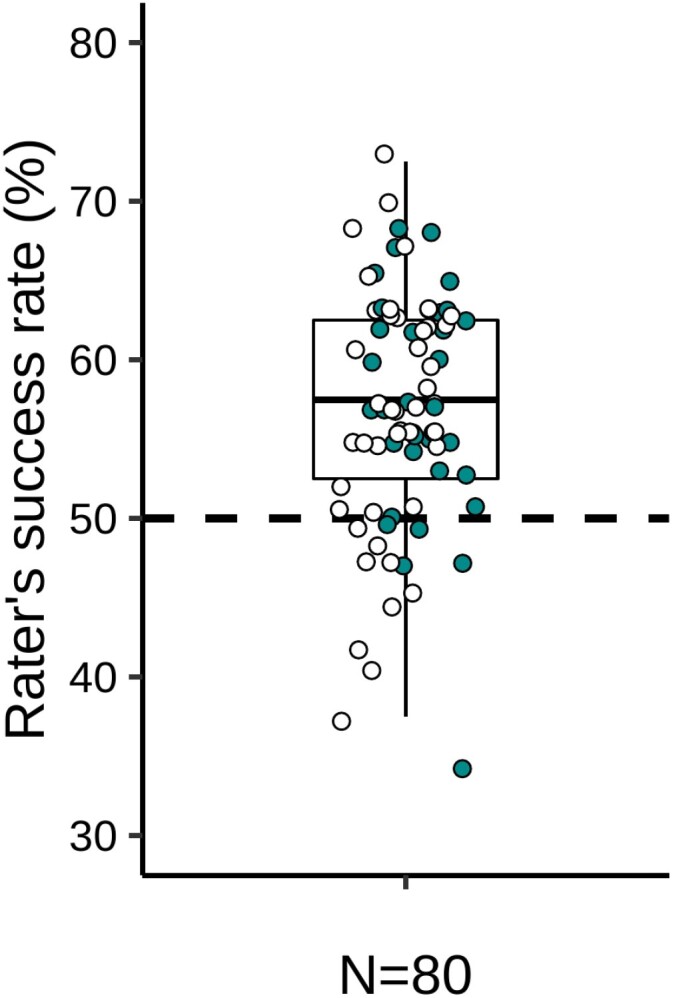
Boxplot representing the proportion of accurate sick odor identifications (i.e. success rate, *N* = 80). The dashed line depicts chance level (50%). White circles represent female raters and green circles represent male raters

The Wilcoxon signed rank test with continuity correction showed that raters were slightly, but significantly, better than chance at identifying sick body odors from paired sick and healthy odor samples (*M* ± SD = 56.7 ± 7.4% versus chance level of 50%, *V* = 2383.5, *p* < .001, effect size *r* = 0.68, Fig. 2).

### Influence of donors’ characteristics on sickness detection

**Figure 3.**
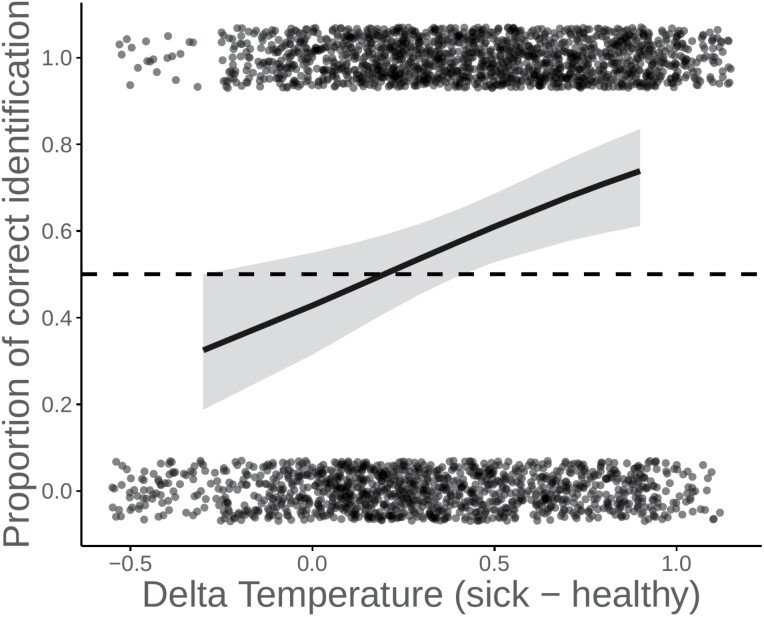
Predicted proportion of correct sick odor identifications as a function of the change in donor temperature excluding the outlier (*N*_Donor_ = 18) between sick and healthy conditions. The slope and 95% CI, as predicted by a GLMM, are shown. The dots represent raw data—i.e. inaccurate (0) and accurate (1) identifications (*N*_obsv_ = 2880 from *N* = 80 raters). The dotted line represents random choice (50%)

Accuracy in the detection of sick individuals was not significantly associated with the increase in donor sickness symptoms (β = −0.05, *SE* = 0.46, *X*^*2*^= 1.08, *df* = 1, *p* = 0.30) or temperature on night 1 of the acute respiratory infection (β = 0.61, *SE* = 0.43, *X*^*2*^ = 1.94, *df* = 1, *p* = 0.16). However, after excluding one donor outlier who had a decrease in temperature of 0.8°C during their infection and was more than −2.5 SD from the mean (i.e. −2.6 SD) (see Supplementary Fig. SM1), we find a significant association between sickness detection and donor temperature (β = 1.48, *SE* = 0.49, *X*^2^ = 9.11, *df* = 1, *p* = 0.003, Fig. 3), suggesting that a greater increase in donor temperature between sick and healthy conditions was related to more accurate sickness detection. It is important to note that temperature increases were small, and only 1 of 20 donors reported having a fever (i.e. temperature above 38°C). Thus, this finding should be interpreted with caution.

### Influence of raters’ characteristics on sickness detection

Accuracy of the raters’ sickness detection scores was not associated with rater characteristics. Specifically, there was no association between the accuracy of sickness detection and rater sex (β = 0.05, *SE* = 0.08, *X*^2^ = 0.32, *df* = 1, *p* = 0.57) or disgust sensitivity measured via the BODS (β = −0.005, *SE* = 0.01, *X*^2^ = 0.13, *df* = 1, *p* = 0.71).

## DISCUSSION

Here, we provide some evidence that humans can distinguish between the body odor of healthy versus sick individuals who have a ubiquitous and commonly occurring natural illness (i.e. an acute respiratory infection) via smell. Our results also suggest that the accuracy of sickness identification increases with a greater change in donor body temperature, but not sickness symptoms. Prior empirical research shows humans can detect olfactory cues to sickness when sickness is experimentally induced by intravenous injection with an endotoxin that causes a transient acute systemic inflammation and flu-like symptoms [12–13]. Although the accuracy of detection is only slightly better than chance levels, our study is the first to provide evidence that humans can detect a naturally occurring acute respiratory infection through olfaction alone.

Animals have evolved complex immune systems to detect and mount defenses against pathogens that enter the body. While highly effective at combating pathogens, mobilizing an immune response is metabolically costly and can cause further debilitating symptoms. Consequently, additional behavioral defenses likely evolved to inhibit contact with disease-causing agents in the first place (for reviews [1, 9]). Evidence of pro-active immune defensive behavior has accumulated across several animal species (for review [33, 34]). For instance, experimental studies with rodents demonstrate that chemical cues of infection (e.g. urine odor) are used to detect and avoid infected conspecifics [18, 33, 35–39]. Rodents easily discriminate between the odors of conspecifics infected versus uninfected with parasites, such as nematodes (e.g. [33, 35, 40]) and viruses, such as influenza [41]. Similarly, healthy social lobsters (*Panulirus argus*) prefer a den with other healthy lobsters over dens containing diseased lobsters [42]. Nearer to humans, observational studies, experiments and chemical analyses have discovered, e.g. (i) mandrills avoid grooming orofecally parasitized conspecifics, (ii) mandrills receive significantly more grooming after being treated for and recovering from orofecally transmitted parasites, (iii) parasites influence fecal odors and (iv) mandrills avoid fecal matter from parasitized conspecifics [43]. Humans also use olfaction to guide decision-making in similar behavioral contexts where smell is demonstrated to be important for other animals, such as the detection and avoidance of microbial and nonmicrobial threats within the environment (for review, [44]); thus, humans too can likely detect olfactory cues to sickness in others.

In the present study, we built on a previous study conducted by our group [24], using the same odor samples; thus allowing us to better compare our findings because we investigated the same disease. This prior study was underpowered with too few raters (*n* = 46), which may have contributed to the non-significant findings. The current study addressed prior limitations by doubling the sample size of raters (*n* = 80) and by using a more sensitive and bias-free method for detecting differences, the two-alternative forced-choice method (2AFC). The 2AFC method lets the observer be guided by any perceptual difference between sick and healthy body odors in their categorization of which of the two body odors is sick and which is healthy. Moreover, the 2AFC method places a smaller demand on working memory, as the odors are presented in quick succession, compared to a sequential presentation procedure, where odors are presented one at a time. The current study also investigates the association between the accuracy of sickness identification through smell and sickness-related changes in donor temperature and symptoms. Here, we do find evidence that humans can discriminate between naturally sick and healthy body odors, albeit only slightly better than chance levels. Our data also suggest that sickness-related changes in temperature, but not sickness symptoms, can contribute to raters’ ability to perceive differences in sick and healthy body odors. Importantly, temperature increases were small, and only 1 of 20 donors reported having a fever (i.e. temperature above 38°C). This finding suggests, similar to other animal species, that humans are able to identify naturally occurring sickness in others through smell, and that it may be related to having a temperature increase. Future research should determine the specific VOCs regulating olfactory detection of respiratory infection. While the magnitude of the effect reported in this study is small, it should be noted that we investigated a single sensory modality. Yet, multisensory cues (i.e. olfactory, auditory and visual) to sickness likely simultaneously influence sickness detection outside the laboratory and in the real world. Indeed, there is evidence that sickness can be detected in faces [10, 11, 22], through biological motion [45] and in body odors [12, 13, 23]. In addition to replicating the current findings, future research should investigate whether the accuracy of sickness detection improves when cues from multiple sensory modalities are observed simultaneously [14].

Rater characteristics, such as sex and disgust sensitivity, may also influence the accuracy of sickness detection. An extensive literature indicates that women typically outperform men on all measures of olfactory acuity (i.e. detection, identification and discrimination) (for meta-analysis [26]) and that women tend to exhibit greater disgust sensitivity than men (for review see [28]). Based on this literature, we hypothesized that women would be better at identifying sick body odors. Yet, we found no evidence that sex was associated with the accuracy of sickness detection. Similarly, we investigated whether greater disgust sensitivity for body odors (measured via BODS) was associated with improved sickness detection rates, but again found no evidence to support this hypothesis. One possible reason for this lack of significant findings is that these effects are too small, and our sample size may not be sufficient to detect the effects of sex differences and inter-individual variation in disgust sensitivity. Alternatively, these characteristics may not affect sickness detection, but rather subsequent approach-avoidance behaviors. In speculation, being female and/or exhibiting higher disgust sensitivity might affect downstream behavioral outputs associated with risk-taking, but not sensory detection of potential sources of harm in the surrounding environment. Thus, now that we have shown humans are somewhat able to detect an acute respiratory infection in others via smell, the next steps should include investigating how this odor information influences decision-making under risk and behavioral outputs, such as social avoidance.

### Study limitations

Sickness induction using LPS strongly activates an immune response and results in both behavioral changes [46–48] and detectable sickness cues [11–13, 22, 45]. In the current study, which used a natural sickness model, raters distinguished between sick and healthy body odors only slightly above chance levels. This small effect may be explained by the fact that donors could suffer from any respiratory virus (with variable expression of detectable cues) and because donors seemed to exhibit a relatively low immune activation, reflected by small changes in body temperature. Still, our results suggest that a change in donor temperature between sick and healthy conditions is associated with greater sickness detection by others via smell. Importantly, we report a significant relationship between sickness detection and change in body temperature only after excluding a statistical outlier who exhibited a higher body temperature when healthy compared to when sick (−0.8°). Therefore, this finding should be interpreted with caution and should be replicated. In fact, one possibility is that any condition that increases body temperature could result in body odor that is perceptually different from the healthy condition. Thus, future research should include an additional control condition, such as heavy exercise, that also increases body temperature.

Moreover, inflammatory markers of infection were not measured in the present study, so we are unable to determine here whether an association exists between circulating levels of cytokines and sickness detection rate. Further, the current study was conducted in the field (i.e. donor homes) rather than in a hospital setting, as in previous studies that used an experimental endotoxemia model. The field approach affords greater ecological validity but less experimental control over donor compliance with study instructions, which may have contributed to relatively weak effects. In addition, the current study reused body odor samples from a previous study [24]. Although evidence suggests freezing preserves body odor samples over time, there is also evidence that repeated thawing can degrade the intensity of samples [49, 50]. Therefore, reusing body odor samples may also result in comparatively weaker odors, and possibly effects.

Finally, the present study may suffer from potential order effects in the sampling of body odor conditions. Due to logistical reasons, donors always participated in the sick condition prior to the healthy condition. Consequently, temporal effects could potentially have confounded results. For example, donors were more familiar with the odor collection procedures when they participated in the healthy condition since they had already participated in the sick condition. This could have caused systematic differences between conditions. Or, perhaps, donors were more fatigued with the procedure by the time they participated in the second condition (healthy), which again could have caused systematic differences between conditions. Because the order of conditions was not randomized, any differences arising from the order in which donors participated in conditions were not ‘averaged out’.

## CONCLUSIONS AND IMPLICATIONS

Our study suggests that humans can detect a naturally occurring acute respiratory infection in others via smell, albeit the magnitude of this effect was small. The size of this effect may reflect the use of a single sensory modality (olfaction), rather than multisensory cues of sickness. In natural social interactions, we receive information about others simultaneously through multiple sensory modalities (visual, auditory and olfactory). Thus, future work should determine whether exposure to multimodal cues of sickness improves sickness detection. In addition, while we found some evidence to indicate that sickness detection is related to an increase in temperature, future research should further investigate involved mechanisms and factors influencing the detection of sickness odor cues, and whether these findings generalize to other contagious diseases, such as Covid-19.

## Supplementary Material

eoad016_suppl_Supplementary_Data

## Data Availability

The data that support the findings of this study are openly available in Figshare at http://doi.org/10.6084/m9.figshare.23051867.
