# Supplementary material for: Humans can detect axillary odor cues of an acute respiratory infection in others
Source: Evol Med Public Health. 2023 May 27;11(1):219–28. doi: 10.1093/emph/eoad016 (PMC10324639; doi:10.1093/emph/eoad016)
Supplement: eoad016_suppl_Supplementary_Data [file eoad016_suppl_supplementary_data.docx]

**Humans can detect axillary odor cues of an acute respiratory infection in others**

Supplementary Data

**Descriptive statistics**

Table SM1. Raters’ characteristics

|  | Mean | SD |
| --- | --- | --- |
| Age | 31.4 | 10.70 |
| BODS | 22.5 | 3.10 |
| Sniffin' Sticks score | 6.0 | 1.45 |

Table SM2. Donors’ characteristics

|  | Condition | Mean | SD |
| --- | --- | --- | --- |
| Temperature | Healthy | 36.55 | 0.08 |
|  | Sick | 36.94 | 0.13 |
| Symptoms ratings | Healthy | 0.8 | 0.24 |
|  | Sick | 11.0 | 0.83 |

**Figure SM1**

While investigating the influence of donors' change in body temperature on sickness detection, one statistical outlier was identified (Figure SM1). This donor had a lower body temperature when sick compared to when healthy, and this negative change in body temperature (-0.8°C) was more than -2.5 SD from the mean (i.e., -2.6 SD). Therefore, the main text reports analyses including and excluding this outlier.


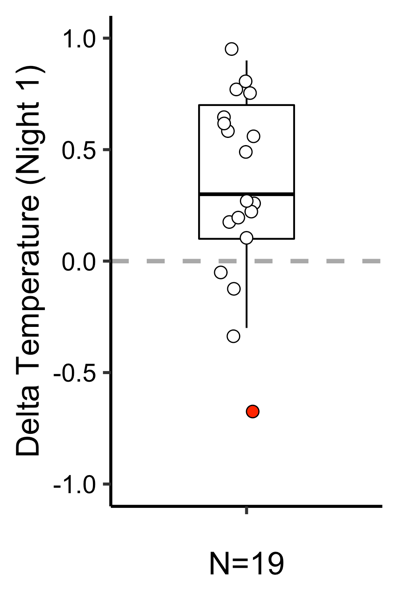


**Figure SM1.** Boxplot representing the change of donors’ body temperature between sick and healthy conditions. A positive value means that the donor had a higher body temperature when sick compared to when healthy. A negative value means that the donor had a lower body temperature when sick than when healthy. One statistical outlier (in red) was identified with a relatively low negative value.
